# Applied research of comprehensive advance geological prediction in Daluoshan water diversion tunnel

**DOI:** 10.1038/s41598-023-36090-8

**Published:** 2023-06-06

**Authors:** Mingqing Liu, Qinyu Gan

**Affiliations:** 1 College of Architecture and Energy Engineering, Wenzhou University of Technology, Wenzhou, 325035 China; 2 College of Design and Art, Wenzhou University of Technology, Wenzhou, 325035 China; 3 Wenzhou Key Laboratory of Intelligent Lifeline Protection and Emergency Technology for Resilient City, Wenzhou University of Technology, Wenzhou, 325035 China

**Keywords:** Natural hazards, Solid Earth sciences

## Abstract

In order to study the accuracy of comprehensive advanced geological prediction methods in tunnel construction projects, this paper takes the Daluoshan Water Diversion Tunnel Project in Wenzhou, Zhejiang Province as the basis of the project, selects a typical section of the water diversion tunnel, and uses Tunnel Seismic Tomography and Ground Penetrating Radar to transmit seismic and electromagnetic waves to the surrounding rock face of the tunnel, and process and interpret the collected signal information. Advanced borehole and drilling techniques are used for verification. The results show that the geological prediction results are consistent with the actual revealed conditions, and the advantages of various technologies can be exerted and mutually verified through advanced geological prediction, which can significantly improve the accuracy of advanced geological prediction in the application of water diversion tunnels and provide reference and basis for later construction, and provide safety assurance.

## Introduction

With the continuous development of China's economy, the construction of highway tunnels, railway tunnels, hydropower projects, and cross-basin water diversion tunnel projects has been accelerated, resulting in tight construction schedules and shortened exploration and design time in the early stages. This has led to insufficient time for detailed geological exploration of the entire tunnel using current survey methods, making it difficult to accurately and comprehensively identify the engineering geology, hydrogeology, and other adverse geological conditions^1,2^. In the actual tunnel construction process, when encountering areas with large geological changes, complex structures, such as karst, fissures, and faults, if relevant disaster prevention and control work is not carried out, it is easy to form geological disasters such as tunnel blockage, deformation, water inrush, and collapse, which can have a huge impact on people's property safety and engineering progress^3–10^.To ensure the safety of tunnel construction, appropriate means are needed to identify the geological conditions early and carry out prevention in advance. As an early detection method, advanced geological forecasting plays an important role, which can obtain information about the surrounding rock ahead of the face in advance and effectively reduce the impact of adverse geological areas on tunnel construction safety^11–13^.

According to the different detection methods and means, existing tunnel advanced geological prediction technologies can be divided into two categories, they are destructive and non-destructive detection^14–17^. Destructive detection mostly uses the method of advance drilling, which conducts drilling work on the tunnel face, analyzes the lithology and structure of the surrounding rock in a certain range in front of the tunnel face through core drilling, and has the advantages of high detection accuracy and intuitive results. However, the limited number of advance drilling information results in high cost, small detection range, and low representativeness of the results. Moreover, since this method is carried out on the tunnel excavation face, it also affects the tunnel construction progress^18,19^, making it difficult to be widely popularized. Non-destructive detection technologies are more varied, such as surface geological survey based on surface geological outcrops and research area geological structures, tunneling machine geological prediction based on parameters such as shield machine cutterhead speed, cutterhead torque, thrust, and advance speed^20–22^, and geophysical detection methods based on differences in physical properties and structural differences of surrounding rock^23–25^. Among them, the tunnel advanced geological prediction technology based on geophysical detection methods is an effective means and main method for guiding tunnel construction methods, reducing tunnel geological disasters, and ensuring normal tunnel construction due to its advantages of fast detection speed, large detection range, and low detection cost.

There are many tunnel advance geological prediction technologies based on geophysical methods, such as the Transient Electromagnetic Method (TEM) tunnel advance geological prediction technology. This method is based on the difference in electric and magnetic properties of anomalous bodies. A pulsed magnetic field is emitted by a non-grounded loop device or grounded line source on the tunnel wall or heading face. The conductive geological body in the target area induces a secondary field under the influence of the pulsed magnetic field. Detection of the water-rich areas is achieved by analyzing this secondary field^26–28^.The geological radar method is also a widely used advance geological prediction technology. This method is applied in the heading face and tunnel wall, using high-frequency electromagnetic waves emitted by the ground penetrating radar antenna. The detection of adverse anomalous bodies such as fractured zones and water-rich areas is achieved based on the difference in dielectric constants of the target anomalous bodies. However, due to the attenuation of electromagnetic waves, the detection range of this technology is relatively shallow in practical applications^29–31^.The infrared detection method based on the difference in radioactive emissions of anomalous bodies is a non-contact detection technology. It detects water-containing geological bodies within a range of 30 m in front of the heading face by identifying and analyzing the infrared field changes formed by the radiation of the surrounding rock mass. In the construction process of tunnels, there exist various metal components such as steel pipe supports and scaffolding, which interfere with the application of the geophysical methods based on electrical and electromagnetic detection^32^. While the infrared detection technology has less impact on tunnel construction, its detection accuracy and results are greatly affected by the humidity and temperature of the detection environment, and are also sensitive to external interference.

Seismic detection technology identifies anomalies in the front of the tunnel face by detecting differences in rock density, velocity, and structure. It has the advantages of high detection accuracy, large detection range, and low electromagnetic interference from metal pipelines. It is currently the main technology for high-precision advance geological prediction in tunnels^33–36^. However, the energy of the source wave generated by the cutter head cutting the rock mass is weak, and the effective signal energy contained in the single-shot received at the surface is weak, making identification difficult. Also, it is difficult to achieve continuous source wave excitation, which is essential for seismic detection in tunnels. As a result, the reliability of the processing results of a small amount of seismic signals is poor. In addition, to improve the detection resolution, seismic interference methods usually require deconvolution processing of the signals received at the surface using the source wave signals. The imaging results of anomalies are greatly affected by the signal-to-noise ratio and phase delay of the source wave signals.

Based on the above problems, this paper conducts research on tunnel advanced geological prediction technology based on seismic exploration. Considering the geological complexity, long route, and large burial depth of the Daluoshan Water Diversion Tunnel, comprehensive advanced geological prediction is used to detect the geological conditions of the surrounding rocks ahead of the tunnel face. Mainly, two geophysical exploration technologies, TST and Ground Penetrating Radar (GPR), are applied to the water diversion tunnel project, and advanced blasting holes and advanced drilling are used for verification. Finally, the effect of comprehensive advanced geological prediction is analyzed and studied.

## Engineering background

### Project overview

The Daluoshan Water Diversion Tunnel is a major water supply line for the westward water plant in Wenzhou, spanning across the Ouhai and Longwan districts. It runs through the Daluo mountain range, with a tunnel length of approximately 8.3 km and a net height of about 6.9 m, forming a gate-like shape. The tunnel is located around 400 m away from the nearby Tianhe East Reservoir, with a height difference of approximately 310 m between the reservoir bottom and the tunnel top. The tunnel is deeply buried and contains abundant water.

### Engineering geological conditions

According to geological investigation and engineering drilling, the main strata of the tunnel section include Quaternary strata: Upper Jurassic Jiuliping Formation (J3j), Upper Jurassic Xishantou Formation (J3x), and intrusive rock. Quaternary strata: erosion and accumulation layers of marine, residual slope, and alluvial fan.

**Figure 1:**
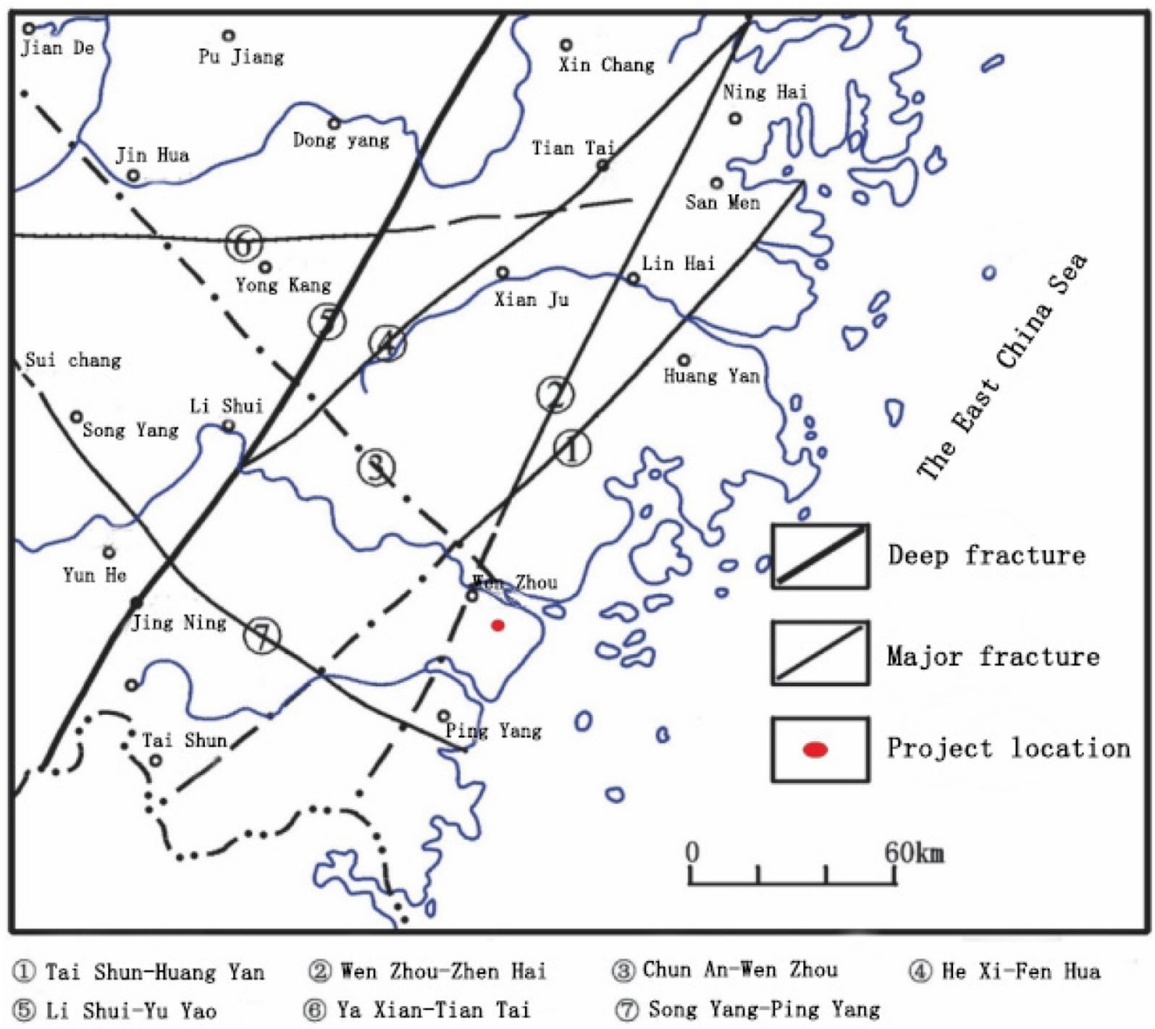
Regional geological structure.

The geological conditions of the tunnel project are relatively complex.The tunnel passes through hills and mountains, and the surrounding rock formations vary greatly. They include porphyritic granite, rhyolite, vitric tuff, quartz-feldspar porphyry, andesite, and granite, all of which belong to hard rocks. The rock mass at the tunnel entrance and exit is mainly of grade V to IV, while the rock mass inside the tunnel is mainly of grade II to III, with the fractured and jointed zones being of grade V to IV. There are regional fractures in the tunnel area, including the northeast-trending Wenzhou-Zhenhai Fault, Taishun-Huangyan Fault, and northwest-trending Chun'an-Wenzhou Fault. The tunnel area has developed grade IV structural joint fissures, which mainly trend in the northeast and northwest directions with dip angles of 75° to 85°. The joint fissures are slightly opened and closed, with a spacing of 0.5 to 1 m near the intruded rock mass, and a spacing of 0.1 to 0.2 m near the local dense area. The fissure surfaces are straight and contain iron and manganese minerals. There is a fault F1 developed in the area, which crosses the tunnel at an angle of about 26° and has a strike of 228°∠ 80° to 85°. The fault is approximately 6.1 km long and has a broken width of 5 to 10 m. The rocks on both sides of the fault are crushed and have developed joints, and the rock mass is broken. The regional geological structure map is shown in Fig. 1.

## Review and introduction of advanced geological prediction techniques

Currently, the most common methods for advanced geological prediction in China are mainly divided into engineering geological analysis prediction method, geophysical exploration method, and advanced drilling method based on the different instruments used. The geophysical exploration method includes various technologies such as sonar, high-density electrical method, transient electromagnetic method, seismic reflection wave method, and geological radar method, as shown in Table 1. In this study, a combination of TST and geological radar techniques for long and short distances was used in conjunction with advanced blast holes and advanced drilling for verification and validation of the prediction method.

**Table 1:** Comparison of advanced prediction methods.

Category	Forecasting method	Method principle	Technical advantages and disadvantages
Engineering geological analysis and prediction method	Geological cataloging	Analyze the engineering geological conditions inside and outside the tunnel	It does not interfere with construction, provides complete data records for later tunnels, and is applicable to tunnels with simple geological structure
	Geological sketch	The physical characteristics of rocks and structural fractures of rock strata revealed by the observation of the face	Short operation time, suitable for simple geological conditions
Advance Boreholes	Advance heading	Parallel to the tunnel or directly excavate small test pits in the tunnel	High accuracy, increased construction cost and time
	Advanced horizontal drilling	Drill hole survey at the face	It can directly reveal the formation conditions and increase the construction time and cost
Geophysical prospecting method	Sonic method	Use the law of sound wave propagation to receive reflected waves	The prediction distance is less than 15 m and the accuracy is poor; Simple operation, less interference from outside
	Electrometric method	Rock resistivity difference	It is simple and convenient, suitable for rock strata with large difference in resistance values, and is easy to be disturbed on site
	GPR method	The difference of rock dielectric constant and the difference of reflected electromagnetic wave to distinguish rock	With high resolution, it can identify geological unfavorable bodies such as rock fault zone, water-bearing zone and cavity. Measuring distance 15 ~ 30 m
	Seismic reflection method (TST, TSP, ISP, TGP, etc.)	When the seismic wave encounters rocks with different wave impedance (rock density and wave velocity), the reflected signal changes	The resolution is low, the prediction length is about 100 m, the surrounding rock can be classified, the preliminary work preparation is complex, and the later data processing results are greatly affected by the technician's experience
	Infrared detection method	The infrared radiation energy signal from the geological body can form a radiation field	The operation is simple and does not affect other construction, and the measurement of tunnel water content is relatively accurate

### Tunnel seismic tomography technology (TST)

1. Principle

**Figure 2:**
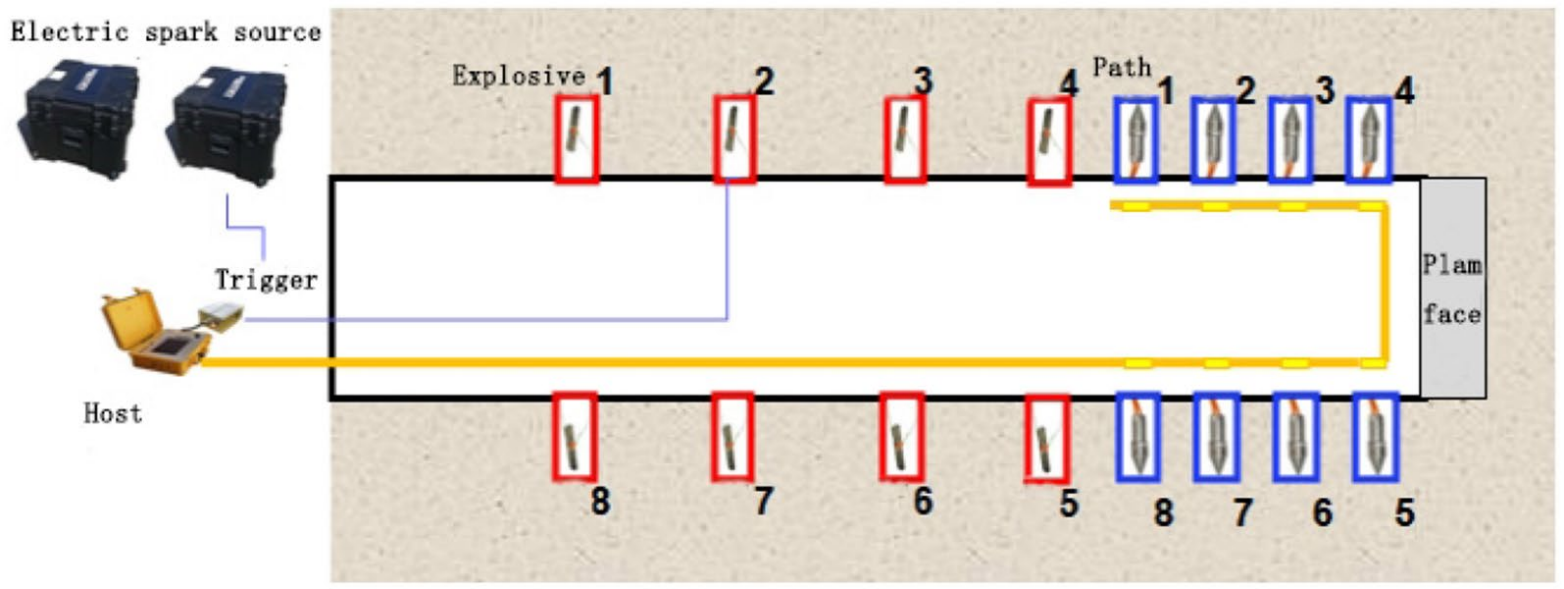
The principle schematic diagram of the TST system.

Tunnel Seismic Tomography is an advanced geological prediction technology for long-distance tunnels. The principle of it is to use the artificial seismic source generated during tunnel excavation. The seismic waves propagate through the underground media via scattering, refraction, reflection, and other multiple modes. Eventually, the reflected waves are received by the receivers on the ground. By imaging based on the received reflected waves and the predetermined geological model, geological structural information can be obtained. It can also be seen in Fig. 2.

(2) Acquisition and processing

**Figure 3:**
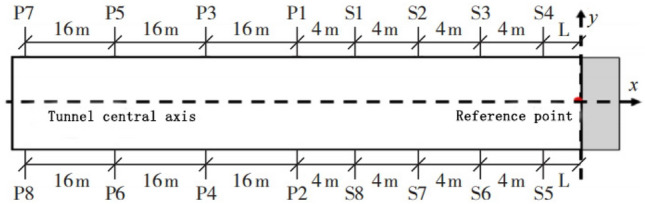
Layout of TST observation system.

The TST observation system in this study adopted a 4 m receiver spacing layout for testing the face of section K2 + 022, as shown in Fig. 3. The main details are described as follows.

1. Eight receivers are placed in the inner walls, four on each side, with a spacing of 4.0 m and a burial depth of 1.8 m.
2. Eight electric spark source holes are arranged in the inner walls, four on each side. The first source hole on each side is located 4 m away from the nearest receiver, and the other three are spaced 16 m apart, with a burial depth of 1.8–2.0 m.
3. Both the receiver holes and the source holes are drilled with a hand-held drill with a drill bit diameter of ø60.
4. Water cannon mud coupling and sealing are adopted.

**Figure 4:**
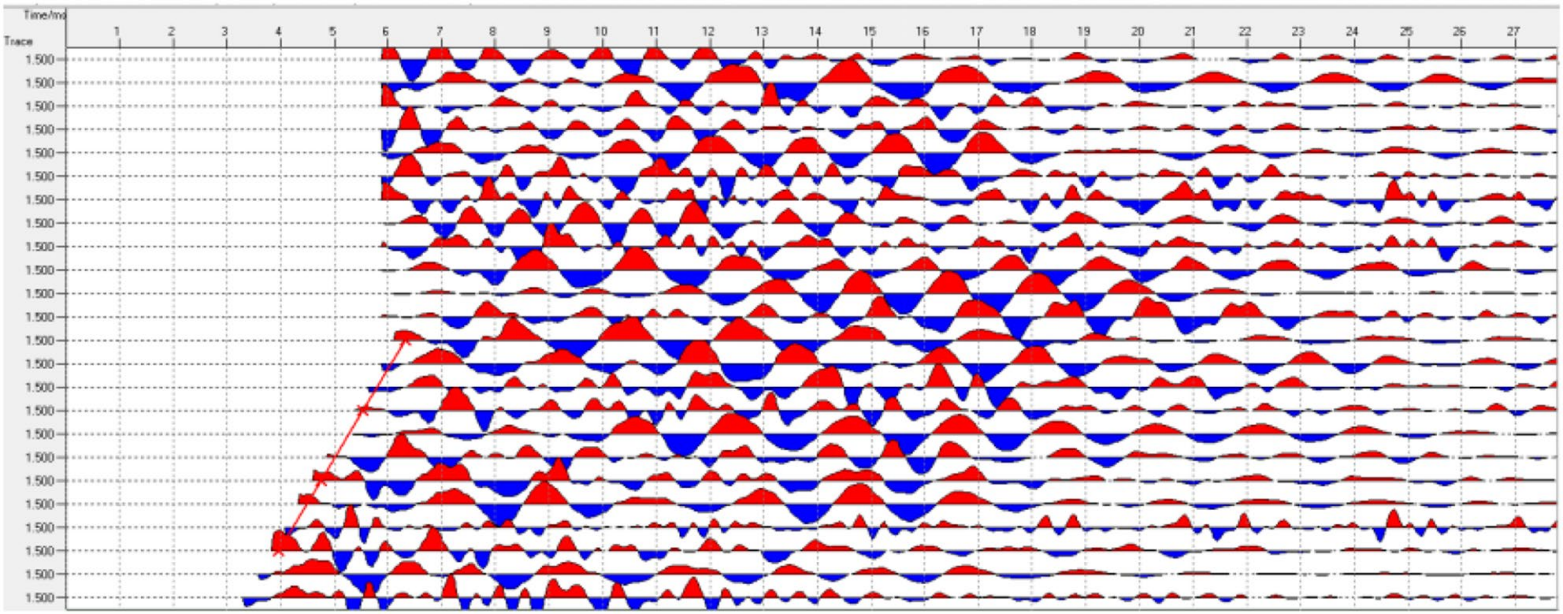
Typical single shot record after processing.

**Figure 5:**
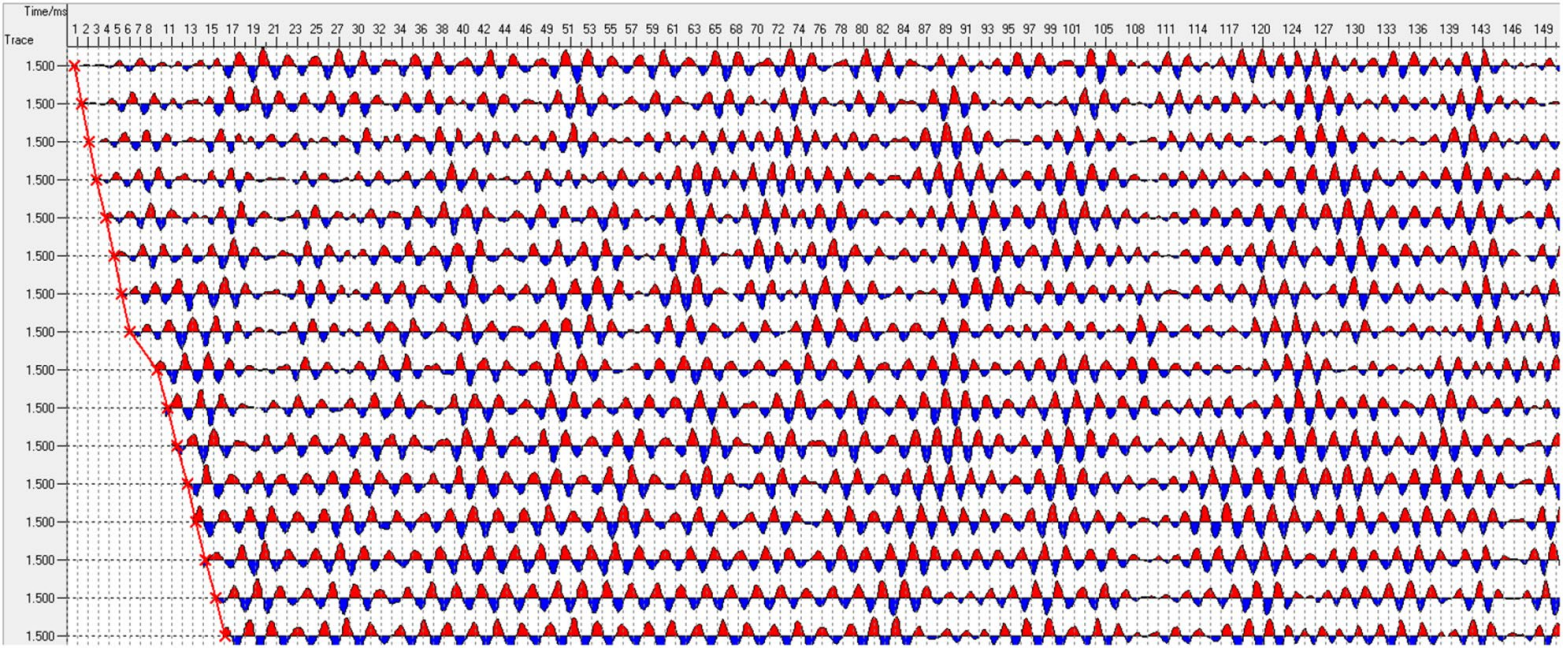
Typical record after wavelength separation.

**Figure 6:**
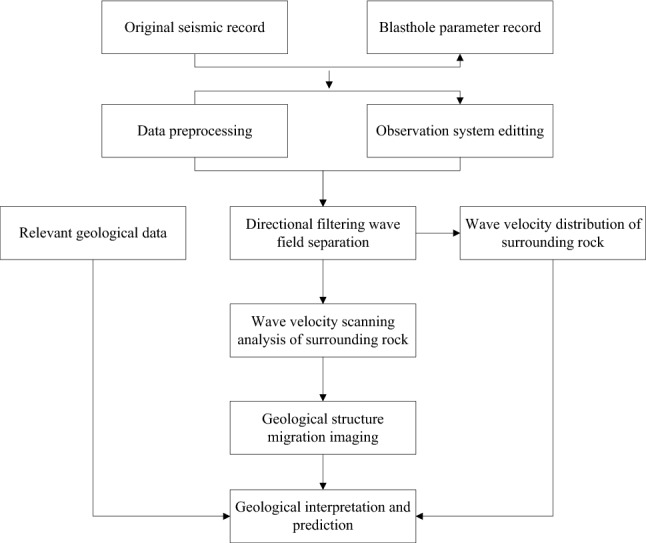
Data processing flow of TST system.

The data collected by the TST is pre-processed to obtain single shot processing results, as shown in Figs. 4 and 5. Then, the seismic wave migration imaging and velocity analysis are obtained through a series of processing steps, including inputting the geometric location parameters, wave field separation, truncation, multi-point gain, and trace normalization. Finally, geological interpretation is performed. The specific process of TST is shown in Fig. 6.

### Ground penetrating radar (GPR)

#### Principles

**Figure 7:**
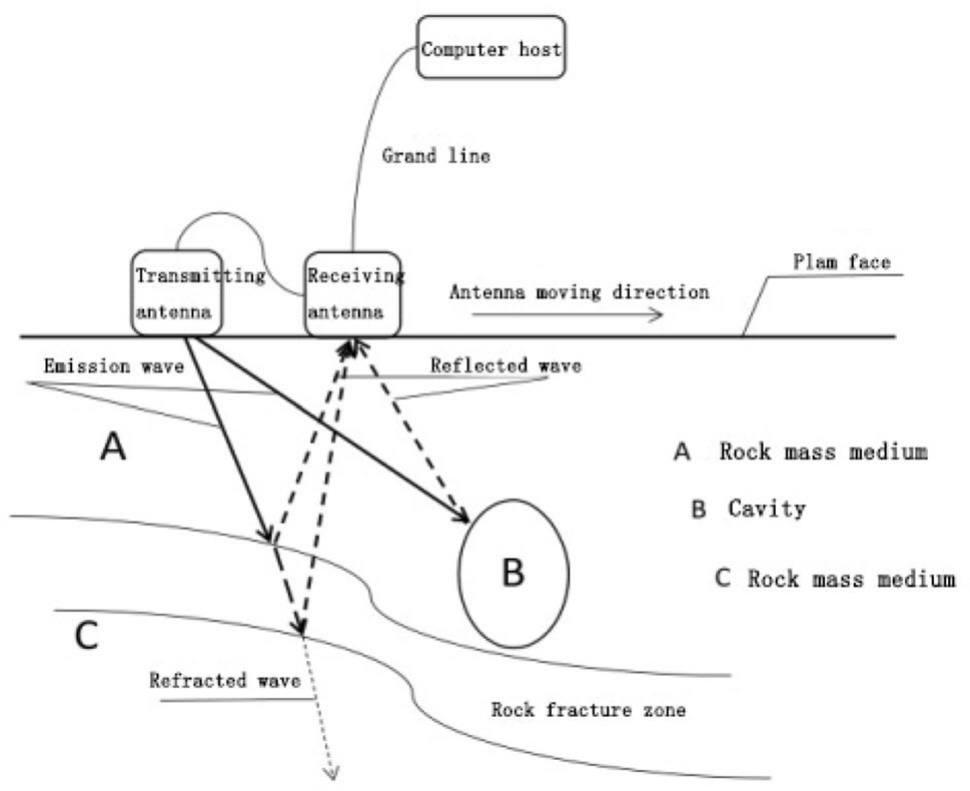
The principle of Ground penetrating radar electromagnetic wave propagation.

Ground penetrating radar (GPR) is a short-range tunnel advanced geological prediction technology. Its principle is to emit high-frequency electromagnetic waves, which are introduced into the ground. When the electromagnetic waves encounter medium interfaces or substances with different dielectric constants, phenomena such as reflection, transmission, and refraction occur. By receiving, recording, and processing these signals, the properties and structure of underground media can be inferred, as shown in Fig. 7.

#### Acquisition and processing

This paper employed the Ground Penetrating Radar (GPR) to collect data from the K2 + 062 section, and performs linear reciprocal-point detection scanning on the rock surface. The data is processed by procedures such as data editing, zero correction, background removal, removal of horizontal signals, band-pass filtering, sliding averaging, and gain adjustment, to obtain the profile results of the GPR survey line position.

**Figure 8:**
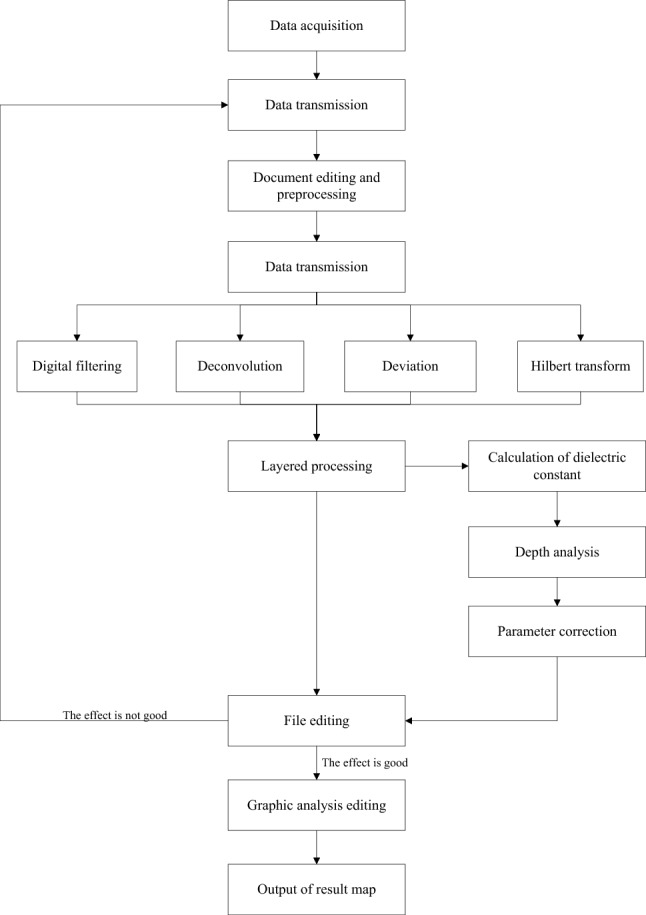
Data processing flow of GPR.

During the point measurement process of geological radar, it is required that the antenna is tightly attached to the coupling palm surface to reduce the impact of multiple reflection waves between air and rock masses. Post-blasting measurement is difficult due to various factors such as unevenness of the palm surface and the presence of excavation equipment and power lines in the tunnel. Generally, a point measurement mode is selected with a distance of 10–15 cm between two measurement points, and at least 80 points are recommended to ensure the imaging analyzability of measurement data in the later stage. Considering the various factors in the tunnel, such as excavation equipment and power lines, a comparison and verification measurement is conducted by using a two-way measurement. The geological radar data processing flow is shown in Fig. 8.

## Analysis of comprehensive geological forecasting results in the test section

**Figure 9:**
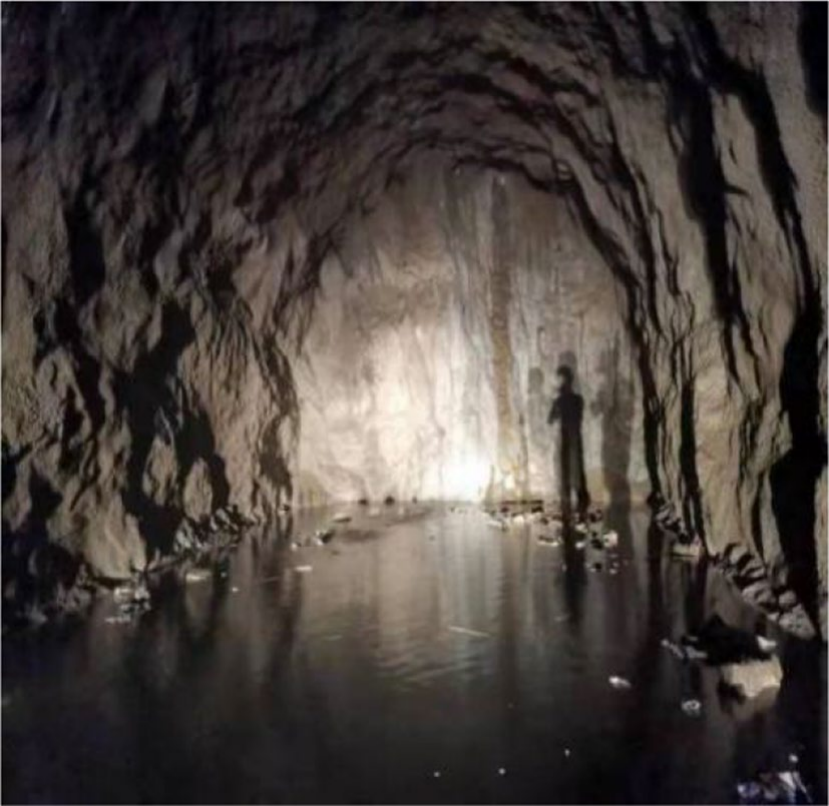
K2 + 077 section face.

The test section is from chainage K2 + 22 to K2 + 122 of the Daluoshan Water Diversion Tunnel. According to the results of engineering geological survey, the main lithology of this section is porphyrite, porphyritic andesite, and crystal tuffaceous welded tuff, all of which are hard rocks. Some areas contain gravel and powdery clay. The surrounding rock of the tunnel section is mainly grade II–III, with dense joint zones of grade V–IV, and weak and fragmented rock masses exist. The tunnel face rock of section K2 + 077 is mainly composed of mudstone and siltstone, with the weathered bedrock as the main foundation. The overall stability is relatively good, the rock mass is weakly weathered, and the joints are developed and embedded, appearing fragmented. The joints are slightly open and closed, with a spacing of 0.3-1 m, extending more than 2 m, and there are local seepage phenomena, as shown in Fig. 9. According to the site conditions, TST advance geological prediction method, geological radar method and advance geological blast hole method are mainly used in this section for advance geological prediction.

### Analysis of TST advance geological prediction results

**Figure 10:**
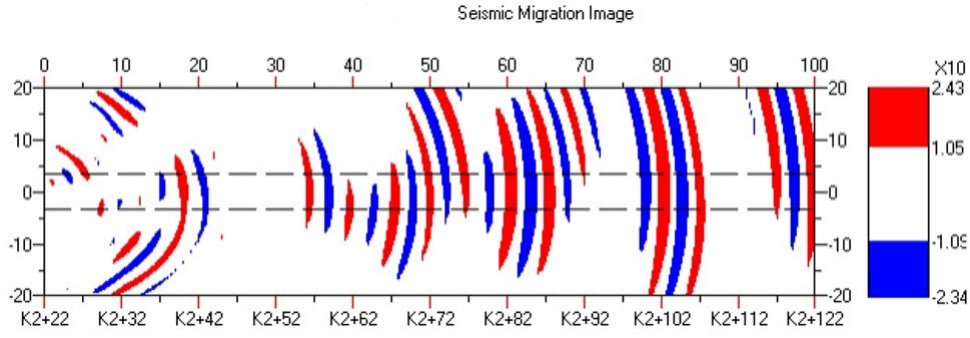
Seismic wave migration imaging.

Figure 10 shows the offset imaging results of the advanced geological prediction for the K2 + 022 to K2 + 122 sections of the Daluoshan Water Diversion Tunnel. The left end of the horizontal axis represents the starting point of the face mileage. The vertical axis of the offset imaging represents the width direction of the tunnel. The red and blue stripes in the vertical wave offset image represent zones with lithological changes. Red indicates that the rock mass has become harder, with an increased wave velocity, while blue stripes indicate the opposite. Alternating red and blue stripes indicate that the integrity of the surrounding rock in this section is relatively poor, and there may be a fractured zone or a weak interlayer.

**Figure 11:**
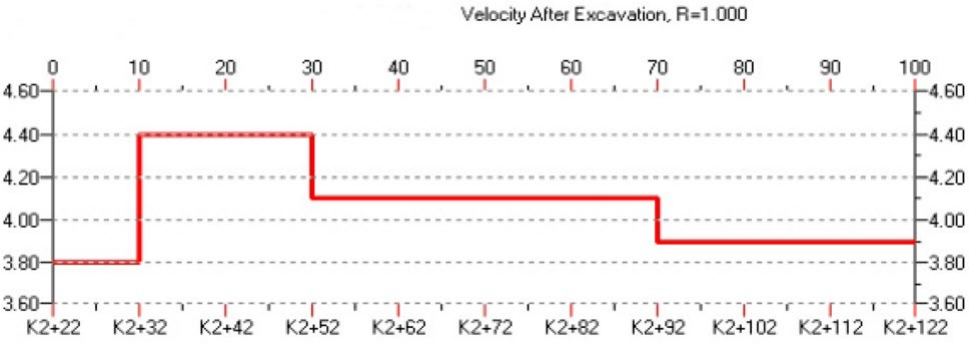
The analysis of P wave velocity.

**Figure 12:**
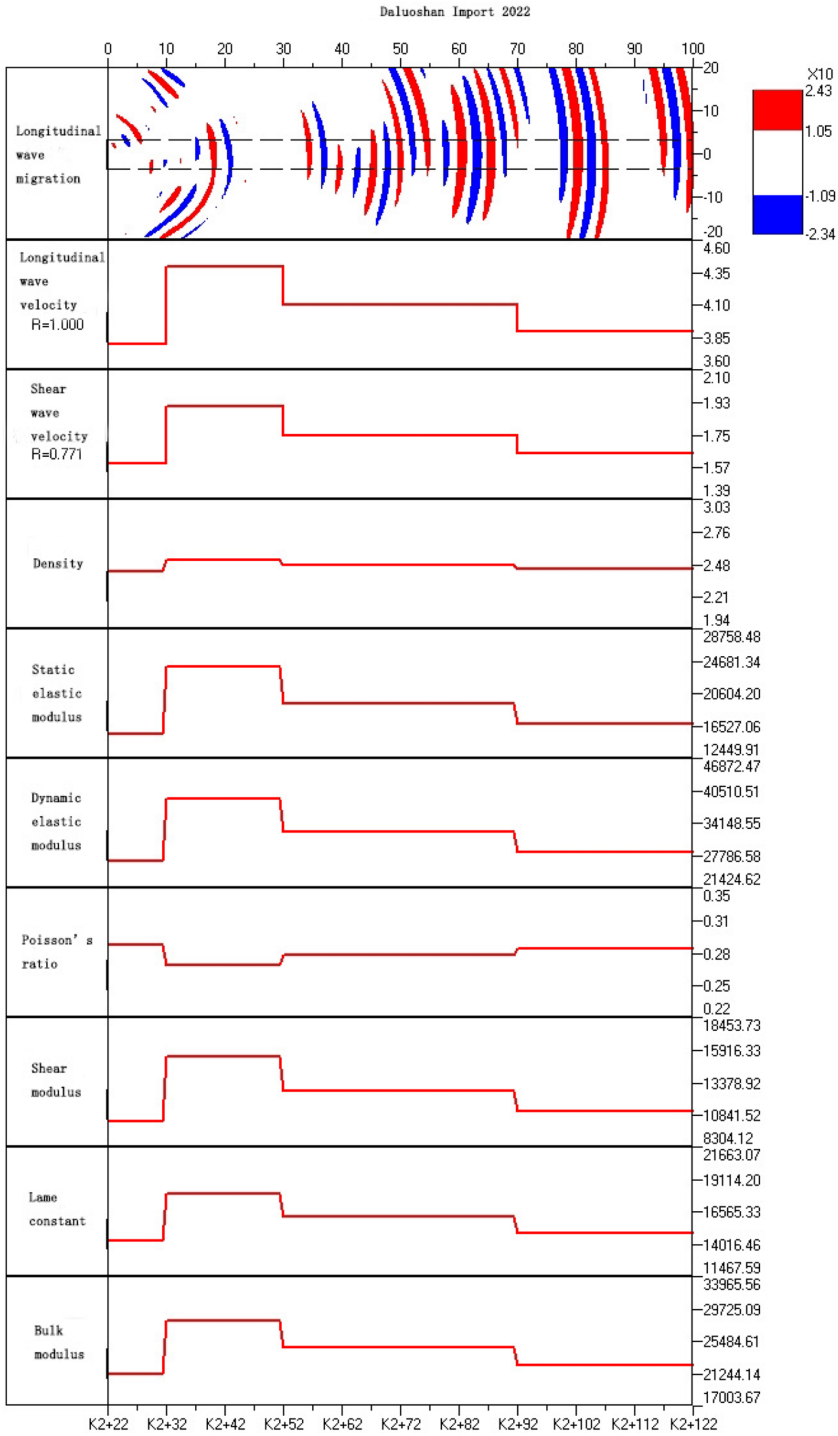
The trend of variation in rock physical and mechanical parameters in the test section.

**Figure 13:**
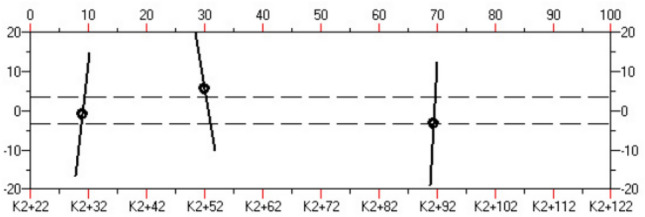
Reflection surface extraction map.

The distribution of rock mechanics properties is reflected by the distribution of rock mass wave velocity. High wave velocity indicates intact and high elastic modulus rock mass, while low wave velocity indicates fractured rock mass with low elastic modulus. The wave velocity image corresponds well with the geological structural image. In the structural offset image, the areas with dense reflection stripes indicate complex structures and developed tectonics, which correspond to low wave velocity zones in the wave velocity image. while areas with few structural stripes indicate uniform and dense rock mass, corresponding to high wave velocity zones in the wave velocity image. According to the analysis of wave velocity (as shown in Fig. 11), the variation of comprehensive physical and mechanical parameters of surrounding rock (as shown in Fig. 12), the reflection surface extraction map (as shown in Fig. 13), and combined with geological data, it can be concluded that the geological situation within 100 m in front of the tunnel face can be roughly divided into three sections as described below.

Section "Introduction": 0 ~ 10 m (K2 + 022K2 + 032)

The length of the surrounding rock in this section is 10 m, with a longitudinal wave velocity of 3800 m/s and low strength. The offset image shows a denser combination of red and blue stripes, which is inferred to be affected by the unloading zone near the face. The integrity of the surrounding rock is poor and its self-stabilizing ability is weak. It is recommended to reinforce the surrounding rock and pay attention to the falling blocks or collapse caused by rock fragmentation during construction.

Section "Engineering background": 10 ~ 30 m (K2 + 032 K2 + 052)

The length of the surrounding rock in this section is 20 m, and the longitudinal wave velocity increases to 4400 m/s, with higher strength than the previous section. The offset image shows slightly fewer red and blue stripes in this section, and it is inferred that the joint fissures in the surrounding rock are less developed, and the rock type is slightly weathered spotted rock with better integrity.

Section "Review and Introduction of advanced geological prediction techniques": 30 ~ 100 m (K2 + 052 K2 + 122)

The length of the surrounding rock in this section is 70 m, with a longitudinal wave velocity of 3900 ~ 4100 m/s and lower strength than the previous section. The offset image shows an increase in the number of red and blue stripes. From 50-70 m, there are red and blue alternating stripes, indicating a higher possibility of the existence of fractured zones or weak interlayers in the surrounding rock, with poor integrity. It is recommended to reinforce the surrounding rock and pay attention to the falling blocks or collapse caused by rock fragmentation during construction.

The above results can be presented in a table, as shown in Table 2.

**Table 2:** Analysis of TST advance forecast results.

Chainage mileage	Length (m)	Wave velocity of rock mass (m/s)	Grade of surrounding rock	Forecast results
K2 + 022 ~ K2 + 032	10	3800	IV	Affected by unloading, the integrity of surrounding rock is poor, relatively broken, and joints are relatively developed
K2 + 032 ~ K2 + 052	20	4400	III	The surrounding rock has good integrity, slightly developed joint fissures and no obvious adverse geological phenomena
K2 + 052 ~ K2 + 122	70	3900–4100	IV	There are many weak structural planes developed and distributed. The surrounding rock is poor in integrity, with fault fracture zone and rich in groundwater

### Analysis of geological prediction results by geological radar method

Radar profile is the basis for interpreting geological radar data. As long as there are electrical differences in the medium in front of the fault surface, corresponding reflection waves can be found in the radar profile.

The recognition of radar profile mainly depends on identifying the common phase axis of reflection wave groups with the same characteristics. Generally speaking, the waveform of the tectonic fault zone on the radar profile reflects a curve that is similar to the trend of the fault zone. The waveform of the weak layer and karst caves is generally composed of many small parabolas that make up a larger area, and there are significant differences with the surrounding wave forms. Practical experience has proved that geological radar has a good reflection on abnormal situations such as water, karst caves, and fault zones in front of the fault surface, but the range of prediction will be relatively shortened. Because the dielectric constant of water is 81, electromagnetic wave energy will be absorbed by water in large amounts, and the detection distance will be relatively shortened. The energy consumption of electromagnetic wave propagation in the formation is also very large, which will also have a certain impact on the detection distance. In addition to finding obvious signal anomalies on the radar profile, the interpretation of the radar image also requires attention to the geological conditions of the construction site of the fault surface and comprehensive judgment based on geological knowledge.

**Figure 14:**
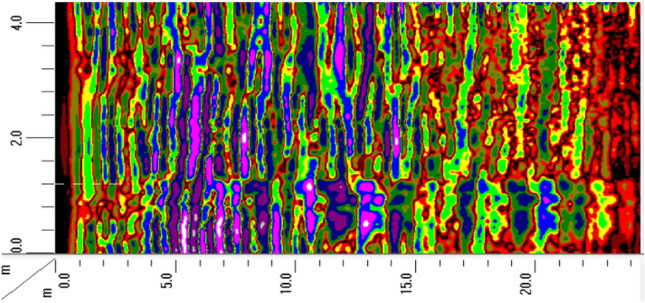
Profile color results map.

**Figure 15:**
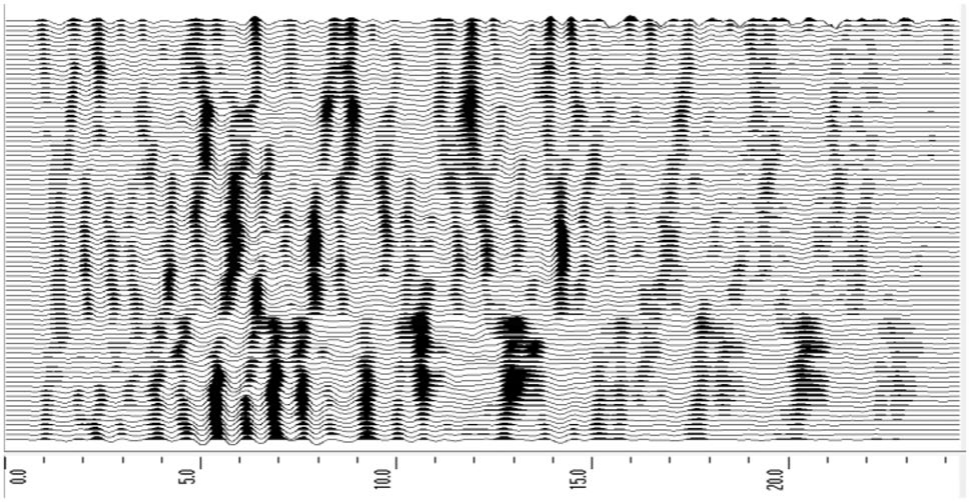
Profile Gray-scale Stacked Image.

Figures 14 and 15 are the color profile and grayscale stack results obtained by the geological radar survey. Based on the observation of signal characteristics such as wave amplitude variation and homogeneity axis, it can be seen that this section has high amplitude, discontinuous homogeneity axis, and obvious high-amplitude characteristics, indicating that there is well-developed groundwater and broken surrounding rock in the measurement range ahead of the tunnel face. Based on a comprehensive analysis of the rock exposure situation, the geological conditions between K2 + 062 and K2 + 087 are obtained and the analysis results are shown in Table 3.

**Table 3:** The analysis results of GPR.

Chainage mileage	Length(m)	Homoaxial continuity	Amplitude change	Analysis results
K2 + 062 ~ K2 + 065	0–3	There is a fault	Low amplitude and small change	The face is affected by blasting, the surrounding rock is relatively broken, the joints are relatively developed, and local water content
K2 + 065 ~ K2 + 077	3–15	Disruption and discontinuity	Large change, high amplitude	The integrity of surrounding rock is poor, the rock is relatively broken, the bedrock fissure water is developed, and the self-stability ability is poor
K2 + 077 ~ K2 + 087	15–25	Not obvious	Low amplitude	The survey section is rich in water and the radar signal is attenuated, so it is impossible to accurately judge the geological conditions

During the exploration, the measuring line was placed 1 m above the ground. The surface of the tunnel face was uneven, causing the radar to jump during the dragging process and preventing it from closely adhering to the surrounding rock. This greatly interfered with the emission and reception of the radar signals. It is recommended to comprehensively analyze and evaluate the development of cracks in the tunnel surrounding rock based on relevant geological data to ensure the quality and safety of construction.

## Comprehensive analysis and verification of advanced geological prediction

### Comprehensive analysis of advanced geological prediction

Combining two advanced geological prediction methods, the comprehensive geological prediction is made for the K2 + 22 ~ K2 + 122 section of the Daluoshan Water Diversion Tunnel. The prediction results can be seen in Table 4.

**Table 4:** Comprehensive geological prediction results.

Chainage mileage	Length (m)	Analysis results
K2 + 062 ~ K2 + 065	0–3	The surrounding rock is relatively broken, the rock mass is weakly weathered, the joints are relatively developed, and the local water content
K2 + 065 ~ K2 + 077	3–15	The integrity of surrounding rock is poor, the rock is relatively broken, the bedrock fissure water is developed, and the self-stability ability is relatively high
K2 + 077 ~ K2 + 087	15–25	The survey section is rich in water and the radar signal is attenuated, so it is impossible to accurately judge the geological conditions

Based on the comprehensive geological forecast, the surrounding rock condition of the K2 + 065 ~ K2 + 077 section of the Daluoshan Water Diversion Tunnel is poor, the rock is relatively fragmented, the foundation rock cracks and water are developed, and the self-stabilization ability is weak. There may be a water-rich broken zone, so during construction, protective measures should be taken to prevent water inrush and other accidents, and drainage work should be done well. The next step is to strictly control the overlap length of the advanced forecast, reduce the blasting cycle footage during drilling and blasting construction, and timely anchor support after excavation to prevent rock instability and collapse.

### Detection and verification of advanced geological blasthole

**Figure 16:**
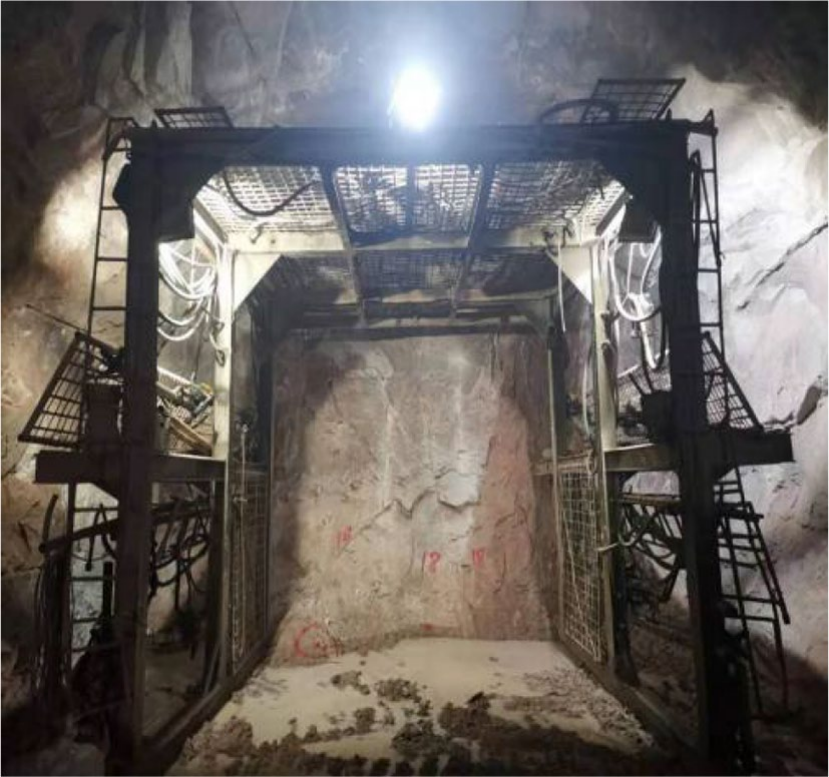
Excavation of the bench face at K2 + 077 in the field.

The tunnel excavation has reached the heading face at the entrance of the Daluoshan Water Dversion Tunnel at the distance of K2 + 065. Ahead-of-face drilling was carried out to verify the accuracy of the geophysical exploration results. According to the ahead-of-face drilling and the actual excavation conditions, the surrounding rock of the tunnel from K2 + 065 to K2 + 077 (where rock bolting support has been installed) was found to be very broken, as shown in Fig. 16, and there were rock blocks falling from the arch. Moreover, there were many water-bearing fractured zones in the tunnel and the water inflow was relatively large. Even after the initial rock bolting, the tunnel still had water seepage. The results of the ahead-of-face geological prediction were consistent with the actual situation. Therefore, it can be seen that the comprehensive use of TST, ground-penetrating radar, ahead-of-face drilling, and other geophysical methods for ahead-of-face exploration of the Daluoshan Water Diversion Tunnel can effectively detect adverse geological conditions ahead of the tunnel heading.

## Engineering application of comprehensive advanced geological prediction construction method

Based on the case of advanced geological prediction for the Daluoshan Water Diversion Tunnel, a comprehensive advanced geological prediction method for tunnels has been established, which combines clear and complete geological analysis with long-distance and short-distance prediction, and can improve the efficiency and reliability of predictions, providing services for engineering construction.

### Characteristics of construction method

1. Based on geological investigation and analysis, and the processing and interpretation of long and short-distance advanced geological prediction data, timely verification of adverse geological bodies ahead of the face through advanced drilling and blasting methods, and identification of potential geological hazards. The complementary advantages of these detection methods not only improve the accuracy of advanced geological prediction, but also ensure the safety of tunnel construction.
2. The comprehensive advanced geological prediction work mode is based on "analysis-prediction method 1-prediction method 2-prediction method 3 and verification-prediction method 4 and checking-summary". On the one hand, it aims to improve the accuracy of prediction results, and on the other hand, it constantly improves the prediction level by continuously revising and supplementing the prediction methods.
3. Different methods of advanced geological prediction have different interpretation principles. By using different geological prediction methods for further interpretation and continuous verification and validation, the interpretation can be constantly revised and improved. With the increase in engineering practice, the results will become more accurate.

### Process of construction method

**Figure 17:**
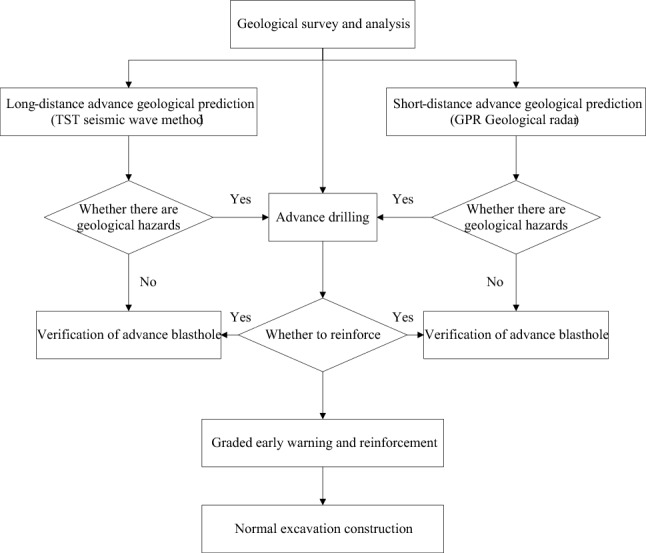
Process flow of advanced prediction technology.

Based on geological observation and analysis, as well as processing and interpretation of long and short-distance advanced geological prediction data, timely verification through advanced drilling and blasting techniques is performed to identify adverse geological conditions such as rock fragmentation, faults, and water-bearing fracture zones ahead of the tunnel face. Possible geological hazards and disaster types are determined, and suggestions for safe construction measures are proposed. Figure 17 shows a set of advanced prediction techniques and procedures applicable to the Wenzhou Daluoshan Water Diversion Tunnel, based on the aforementioned advanced geological prediction technology.

### key operational steps of construction method

(1)Advanced drilling method
①During the drilling process, dynamic control and management should be carried out. The principle of adjusting the drilling depth in real-time according to the borehole conditions should be followed to achieve the prediction purpose.
②When continuous drilling is required, generally 30-50 m can be drilled per cycle, and when necessary, deep boreholes of more than 100 m can also be drilled.
③Different drilling depths are adopted for different sections and purposes.
④For continuous prediction, the front and rear two cycles of boreholes should overlap by 5-8 m.

(2)Advanced borehole blasting method.

3–10 deep blast holes with a length of 5 m are arranged according to requirements for each row of blasting holes, and they are appropriately increased in special sections. They are mainly arranged around the contour and implemented with an outward inclination angle of 30–40° to predict the strata and groundwater ahead of the contour.

(3)TST Advanced geological prediction method.

- ①The layout of the shot points and geophones should satisfy the requirements of wave velocity analysis, directional filtering, and reducing the interference of surface waves.
- ②The geophones should be installed correctly to ensure the quality of seismic waves.
- ③The shot points should be installed tightly and sealed properly to ensure the efficiency of seismic wave excitation.
- ④The blasting machine and trigger line should be in good contact to ensure correct data recording.
- ⑤On-site measurement and recording should be accurate to ensure calculation accuracy.

(4)Ground penetrating radar method

- ①The operational space of the GPR system should be ensured and nearby interference should be avoided.
- ②The connection lines and circuits should be in good contact, reasonably arranged to ensure normal data reception.
- ③Suitable and high-quality materials and equipment should be selected and installed correctly.
- ④The receiver should be closely coupled with the borehole wall to ensure correct reception.

## Conclusions

Taking the Daluoshan Water Diversion Tunnel advanced geological prediction project as an example, the feasibility of advanced geological prediction was verified and validated through observations of the internal and external tunnel conditions, using a comprehensive geological investigation and analysis as the basis, as well as the use of two types of geophysical exploration techniques (TST and geological radar) that were combined at long and short distances. The advanced geological prediction was also verified and validated by the use of advanced blasting holes and advanced drilling. The following conclusions were drawn.

1. To address the geological conditions in front of the heading face during the construction of the Daluoshan Water Diversion Tunnel, a combination of TST seismic wave reflection method, ground-penetrating radar method, and advanced borehole methods were used to carry out comprehensive advanced geological prediction. The more typical sections were selected for analysis, and the adverse geological bodies in front of the heading face of the Daluoshan Water Diversion Tunnel were successfully predicted.
2. Using a single method for advanced geological detection in the field may result in partial signal loss and ineffective prediction due to long distances and energy attenuation. Therefore, a combination of long and short-range prediction methods is necessary to improve prediction accuracy, with mutual verification and validation through the use of advanced boreholes or drilling techniques. This approach provides an effective solution for advanced tunnel prediction.
3. The summary of the prediction of tunnel geological adverse bodies using TST and geological radar shows that TST has good prediction effect on large faults, fractured zones, and soft rock masses in long-distance prediction, but cannot accurately determine the specific location and size of the fault. Geological radar is suitable for predicting underground geological conditions such as fractured rock masses, groundwater-rich zones, and cavities in short-distance prediction.

## Data Availability

The data used to support the findings of this study are available from the corresponding author upon request.
